# A qualitative exploration of the perceptions and information needs of public health inspectors responsible for food safety

**DOI:** 10.1186/1471-2458-10-345

**Published:** 2010-06-16

**Authors:** Mai T Pham, Andria Q Jones, Jan M Sargeant, Barbara J Marshall, Catherine E Dewey

**Affiliations:** 1 Department of Population Medicine, University of Guelph, 50 Stone Road East, Guelph, Ontario, N1G 2W1, Canada; 2 Centre for Public Health and Zoonoses, 103 MacNabb House, Ontario Veterinary College, University of Guelph, Guelph, Ontario, N1G 2W1, Canada; 3 Centre for Food-borne, Environmental and Zoonotic Infectious Diseases, Public Health Agency of Canada, 120-255 Woodlawn Road West, Guelph, Ontario, N1H 8J1, Canada

## Abstract

**Background:**

In Ontario, local public health inspectors play an important frontline role in protecting the public from foodborne illness. This study was an in-depth exploration of public health inspectors' perceptions of the key food safety issues in public health, and their opinions and needs with regards to food safety information resources.

**Methods:**

Four focus group discussions were conducted with public health inspectors from the Central West region of Ontario, Canada during June and July, 2008. A questioning route was used to standardize qualitative data collection. Audio recordings of sessions were transcribed verbatim and data-driven content analysis was performed.

**Results:**

A total of 23 public health inspectors participated in four focus group discussions. Five themes emerged as key food safety issues: time-temperature abuse, inadequate handwashing, cross-contamination, the lack of food safety knowledge by food handlers and food premise operators, and the lack of food safety information and knowledge about specialty foods (i.e., foods from different cultures). In general, participants reported confidence with their current knowledge of food safety issues and foodborne pathogens. Participants highlighted the need for a central source for food safety information, access to up-to-date food safety information, resources in different languages, and additional food safety information on specialty foods.

**Conclusions:**

The information gathered from these focus groups can provide a basis for the development of resources that will meet the specific needs of public health inspectors involved in protecting and promoting food safety.

## Background

Foodborne illness represents a significant health burden in the province of Ontario, Canada. According to an Ontario Ministry of Agriculture, Food and Rural Affairs (OMAFRA) analysis by Campbell (2002), there are more than 2.5 million cases of foodborne illness each year in Ontario, resulting in 9,319 hospitalizations and 135 deaths [1]. Since many cases of foodborne illness are often not reported, the actual number and impact of foodborne illnesses in Ontario is likely to be greater. A study estimating the rate of underreporting for infectious gastrointestinal illness in Ontario found that for each reported case of enteric illness, an estimated 313 cases of infectious gastrointestinal illness occurred in the community [2].

In Canada, food safety is a shared responsibility between the federal, provincial/territorial, and municipal levels of government. The federal government has the principal responsibility for identifying health risks associated with the food supply, assessing the severity and probability of harm or damage to the population, and developing national strategies for managing food safety risks [3]. Each provincial and territorial government has a public health mandate, which includes food safety surveillance, investigations, and compliance [3]. Ontario's food safety program is delivered at the municipal level by 36 organizationally distinct health units that serve the population within its geographic borders. Local public health inspectors (PHIs) play an important frontline role in protecting the public from foodborne illness through their various duties, including inspecting approximately 80,000 food premises [4] across the province to ensure compliance with the Food Premises Regulation 562, R.R.O., 1990, providing safe food handling training to food handlers, and investigating cases of suspected foodborne illness.

While there is an abundance of food safety information and resources available, the information is disseminated over a variety of knowledge media, including textbooks, scientific publications, fact sheets, and websites. In some instances, these resources are not available in a format that is usable and readily accessible to PHIs, or the information is not targeted to their needs. Thus, there may be a need for relevant food safety information materials specifically tailored to the needs of PHIs. In-depth knowledge of PHIs' perceptions of key food safety issues and information needs can better enable the development of resources that will provide enhanced support to PHIs involved in protecting and promoting food safety.

The purpose of this study was to investigate the perceptions and needs of PHIs from the Central West region of Ontario using focus groups. Focus groups are a qualitative research method that are useful in generating rich, detailed data that cannot be acquired via the use of quantitative surveys alone, and help to identify the needs and issues of participants, in a comprehensive, in-depth manner [5,6]. The focus groups were designed to explore (1) the key food safety issues and pathogens of concern to PHIs, (2) the level of confidence that they have in their food safety knowledge, (3) their opinions on the food safety information and resources currently available, (4) the food safety information and resources that they want and need, and (5) what they would like to see in an educational workshop. The Central West region of Ontario is comprised of seven public health regions: Brant County Health Unit, Haldimand-Norfolk Health Unit, Halton Region Health Department, City of Hamilton Public Health and Social Services, Niagara Region Public Health Department, Region of Waterloo Public Health, and Wellington-Dufferin-Guelph Public Health [7]. The region consists of a mix of rural and urban communities, and a population of approximately 2,337,129 people [8].

## Methods

The Research Ethics Board at the University of Guelph approved the study and all participants provided written, informed consent. Between June and July 2008, we facilitated four focus groups with certified PHIs from four local health units. The four health units were purposively selected from the Central West region of Ontario. The inclusion criteria for study participation were that participants have a Certificate in Public Health Inspection (Canada), work in the area of food safety, and speak English fluently.

Focus groups were conducted using a questioning route to standardize qualitative data collection. The questioning route was developed according to Krueger & Casey (2000), and used a series of open-ended questions and pre-planned probes to enhance detail and understanding throughout the discussions. One trained interviewer facilitated each session as a means of ensuring consistency across the four focus groups. The discussions were audio-recorded to ensure transcription accuracy and an assistant recorded notes of the discussion and group interactions. As a means of assessing data accuracy, the interviewer provided a short oral summary of the discussion at the end of each focus group session, after which participants were encouraged to comment to clarify any discrepancies.

The audio-recordings were transcribed verbatim by a professional transcription service. The transcribed texts were then verified using the audio recordings and field notes to ensure that they were correctly transcribed. In order to minimize potential bias introduced in analyzing and interpreting the focus group data, a data analysis procedure was used. The methodology was based on Krueger and Casey's (2000) 'long-table' approach [6,9-11]. Major coding categories were derived from questions in the questioning route, and sub-categories were derived using data-driven content analysis; thus, the data codes were inductively generated from themes that emerged from the data. Direct quotations from focus group participants were used to support and demonstrate themes, and parts of quotations that needed clarifying were supplemented with additional text that was placed within square brackets.

## Results

### Participants

A total of 23 PHIs participated in four focus group sessions. The number of participants in each focus group ranged from four to eight individuals, and each session was between 1.5 and 2 hours in duration. Nine participants were male and fourteen were female. Participants' length of employment as a PHI ranged from one year to thirty-five years, with a mean of thirteen years.

### Food safety issues of concern to public health

When participants were asked to discuss what they considered to be the key food safety issues of concern to public health, the following five themes emerged from the discussions: time-temperature abuse, inadequate handwashing, cross-contamination, the lack of food safety knowledge by food handlers and food premise operators, and the lack of food safety information and knowledge about specialty foods (i.e., foods from different cultures).

#### Time-temperature abuse

Issues related to time-temperature abuse, including insufficient cooking temperature and improper hot-holding, cold-holding, and cooling, were frequently cited as food safety issues by participants. Some participants attributed time-temperature abuse of food to a lack of understanding by food handlers about the proper temperatures for maintaining food safely ("Why does it have to be 4 degrees [Celsius]? They don't understand."). Similarly, another participant commented that some food handlers did not understand the need to handle food properly before being cooked because they believed that the cooking process would eliminate any potential food safety risks that may be present in the food.

#### Inadequate handwashing

The issue of handwashing, which includes insufficient handwashing, improper technique, and the lack of proper handwashing facilities, was described as a key issue by some participants. One participant stated that lack of handwashing by food handlers is "one of the biggest complaints that comes from the public." Another participant commented that, "one of the biggest things I find with handwashing is they don't do it properly." While most participants agreed that inadequate handwashing was a key issue, some participants had different experiences with the issue when it came to food premise inspections. On one end, some participants spoke about instances of food handlers not washing their hands even in their presence and having to tell the food handlers that they forgot to wash their hands. On the opposite end, other participants said that handwashing was not often addressed during food premise inspections because food handlers tend to be more diligent with washing their hands in the presence of a PHI.

#### Cross-contamination

Cross-contamination was frequently raised as a key food safety issue by respondents, and was ranked as one of the top three food safety issues in all four focus groups. While cross-contamination was repeatedly cited as an important issue throughout the discussions, the topic was not elaborated on to any great extent with further comments or discussion.

#### Lack of food safety knowledge

Many participants reported a lack of food safety knowledge by food handlers and food premise operators as a key food safety issue. Most participants felt that many food handlers lacked knowledge about proper food handling practices and why they need to handle food properly. One participant commented, "I think the lack of the basic understanding of food safety is one of the biggest issues that we're dealing with..." On the other hand, a few participants thought that some food handlers have a basic level of food safety knowledge, but are unable to make the link of how to apply their food safety knowledge on a day-to-day basis. For example, one participant spoke of a food premise where for weeks, food handlers recorded elevated temperatures in a refrigerator. Despite knowing what the temperature of the refrigerator should be, no one took any action to address the issue. The participant stated, "there was no connection between checking the temperature and doing something about wrong temperatures." A few participants also discussed how some food premise operators do not understand the requirements of the Food Premises Regulation, and the rationale behind them.

#### Lack of information and resources on specialty foods for PHIs

A lack of food safety information and resources on specialty foods (i.e., foods from different cultures) was said to be an emerging issue by many participants ("We weren't dealing with these things ten years ago, whereas we're dealing with them now."). Participants noted that there are many different types of food available, and described encountering foods with which they were unfamiliar and lacked adequate knowledge. Several participants were in agreement when a participant commented that "just because [a food] is a little different than what we're used to, doesn't necessarily mean it's not safe." Adequate food safety information about specialty foods was said to be lacking and that some foods were not addressed in the legislation (e.g., "There's no cooking temperature for emu in the Food Premises Regulation").

### Other key issues

#### Communication challenges

Several participants spoke of challenges in being able to adequately communicate with food premise operators and food handlers because of language barriers and/or different education levels. For instance, one participant commented on the difficulties that PHIs may face in effectively communicating with people who have limited education or for whom English is not their first language, and indicated that "language is a huge, huge issue". Some participants also perceived that challenges relating to language barriers were becoming more commonplace as the area population expands and becomes increasingly diverse. This theme was well summarized by one participant who said, "I think the knowledge of how to communicate...and how different people communicate differently is almost just as important in this job as the knowledge about the job itself."

#### Lack of consistency

Several participants cited lack of consistency between PHIs, between health units, and between provincial and federal requirements, as issues. Participants commented that the interpretation of the requirements of the Food Premises Regulation may vary between PHIs and/or health units. One participant explained, "we're all different and the regulation isn't detailed enough to really make everybody do things in the same way." Another participant added, "there are a lot of grey areas and there's a lot of different interpretations of those grey areas. And what one health department will interpret, another health department may interpret totally differently." Participants also discussed how this lack of consistency between health units can be difficult for food premise operators that have food establishments in more than one health unit ("so if they have a restaurant in one area and another restaurant in another area, [and] the two health units are not compatible, then they are under different rules and regulations.").

In addition, some participants also spoke about a lack of consistency between provincial and federal requirements. For instance, one participant noted that some of the minimum cooking temperatures required by the Canadian Food Inspection Agency (CFIA; the federal agency responsible for food safety) for certain hazardous foods are different from those in the Ontario Food Premises Regulation, "if you go to the CFIA website, their...recommended temperatures are different."

#### Problems with developing in-house resources

Participants spoke about issues stemming from health units producing their own food safety resources. First, participants discussed the challenges involved with producing resources in-house, and consequently, how it can be a very slow-moving process. One participant explained, "It's become a bureaucratic nightmare within the health unit to develop material." Second, with each health unit producing their own resources, participants said that there is a lack of consistency between the resources produced ("We have all kinds of materials that come out, each health unit does their own thing."). These difficulties were also said to be compounded by some health units not sharing their resources with other health units ("some health units won't share their resources and we have to pay for them, which is brutal.")

### Concern with foodborne pathogens

When participants were asked to list the foodborne pathogens they considered to be of concern to food safety, the responses could be divided into two major categories: (1) those that responded they were concerned with all pathogens that could be present in food, and (2) those that responded they were more concerned with safe food handling practices that reduce the risk of pathogens in general, than on any particular foodborne pathogens. Among the first group, participants said that all pathogens were of concern to them, and one participant explained, "When we're doing an inspection, we're looking at everything that would be pathogenic...[We're] focusing on all environmental pathogens that could be there." While all pathogens were of concern to them, a few participants noted the foodborne pathogens of most concern to them were: *Salmonella*, *Campylobacter *and *Escherichia coli*. Other foodborne pathogens that were also mentioned included: *Listeria*, Hepatitis A virus, Norovirus, and *Yersinia*.

Among the second group, participants were generally more concerned with food handlers taking proper food handling precautions, than with any particular foodborne pathogen. One participant explained, "It's more about washing your hands and cooking the food at the proper temperatures, and addressing cross-contamination...When we're inspecting a place, I don't really focus so much on actual pathogens." However, some of these participants did make one notable exception; a specific pathogen would be of focus to them if it had recently been implicated in foodborne illness or recently been highlighted in the media. As one participant explained, particular foodborne pathogens may become a topic of discussion with food handlers and/or operators during a food premise inspection if, "it's the organism of the day, if it's in papers or if you have outbreaks, then that will surface."

### Confidence with knowledge of food safety issues and pathogens

Most participants said they felt confident with their knowledge of food safety issues and foodborne pathogens. For instance, one participant said, "Food safety is definitely the area of public health that I feel most comfortable with." The only area where some participants expressed less confidence with their knowledge was with certain specialty foods, such as exotic meats, balut eggs, and ceviche.

### Information and resources currently available

Many participants said that they had access to a wide variety of food safety information and resources, including: educational videos, reference books, websites, seminars, and e-mail updates. One participant stated, "...a large bulk of what we do is food safety, is restaurant inspection, so that's one area probably where we do have a fair bit of information available." Several participants spoke of having a number of in-house resources available to distribute to the public and/or food premise operators, such as posters, signs, magnets, fact sheets, and pamphlets.

Several participants also viewed fellow PHIs as a resource for food safety information. Participants in one focus group spoke highly of monthly meetings at their health unit that provide a forum for discussing issues, asking questions, and sharing comments and suggestions. Beyond their own health unit, many participants spoke of contacting more urban health units for information about specialty foods because of their knowledge and years of experience in dealing with these foods. As one participant explained, "Why recreate the wheel if we know they [have] dealt with them?" A few participants also sought information from other PHIs on the Canadian Institute of Public Health Inspectors (CIPHI; the professional organization that represents public health inspectors across Canada) on-line discussion board.

When asked where they obtained food safety information, participants mentioned a variety of online sources, including websites from: other health departments (e.g., Kansas Department of Health and Environment), government agencies (e.g., CFIA, Centers for Disease Control and Prevention (CDC)), and academic institutions. However, other than stating their existence, these sources of information were not largely elaborated upon. For topics in which reliable food safety information were not yet available (e.g., some specialty foods), some participants said they turned to search engines such as Google for any information they could find, with a preference being from reputable sources ("I try to go to the most reputable source [I] can find.")

### Information and resources that are needed

Participants were asked what they thought would help make their job more effective and enhance food safety. The following themes arose during the discussions:

#### Central source for information

In reference to all of the food safety resources currently available to them, one participant said, "So we have to check all of these areas sometimes to find something. Which isn't that bad, but it can be time-consuming." Several participants said that they wanted a central source for accessing food safety resources, which would link PHIs to the most up-to-date information and literature. Some thought that this resource should be provided by the provincial government, CIPHI, or an academic institution involved in public health research. In reference to an academic institution providing this resource, one participant said, "they have access to all the information...they're up-to-date, it's accurate, it's in line with what needs to be out there. And if it's coming from academia... you're not worried about corporate views or things like that coming in play."

#### Resources universal across the province

Many participants said they wanted food safety resources that are universal across the province. For instance, one participant stated, "We've got 32 [*sic*] health units, let's be uniform, the same information, same videos, same thing on handwashing, and same thing on hot-holding and cold-holding. Let it be uniform across the province and by golly we'll see changes." Several participants said that they wanted the resources to be provided by the provincial government, as one participant explained, "Best and most convenient always is coming from the province because there is a consistent message and it's province-wide. It goes to all health units and we can use it as a resource."

#### Latest food safety information

Some participants said that they wanted a source for accurate and up-to-the-minute food safety information to support PHIs in their capacity to make decisions and in providing guidance to the public. Specifically, participants said they wanted food safety information about new specialty foods, background information on foods newly associated with foodborne illness, and information about emerging pathogens and issues ("We know about *Campylobacter *and *Salmonella*, but what's emerging? How do we prevent these emerging things?"). Possible delivery methods that were discussed by participants included an e-mail newsletter or a website with electronic fact sheets ("A website is always easy because it's quick. Because then we could actually go grab a copy if we know where to find it and print it off."). Several participants also stated that some of the food safety resources currently available are outdated and may need some updating. Another participant thought that a process should exist where resources are regularly reviewed to determine whether they need to be updated.

#### Information about specialty foods for PHIs

A need for more food safety information about specialty foods was a common theme throughout the discussions. Some participants commented that additional information would better enable PHIs to assess the safety of these foods. For instance, one participant recounted an experience in which she encountered a specialty food being held at room temperature at a food premise. Being unfamiliar with the food, she resorted to cutting open the food to assess whether it was shelf stable or required refrigeration. She added that she would have been better able to make that assessment had more information on that type of food been available. The types of information that participants wanted about unfamiliar specialty foods included: ingredients, required treatment, shelf stability, and pictures or diagrams of the food.

#### Resources in different languages

Several participants said they wanted food safety resources available in different languages to distribute to food handlers or food premise operators. For instance, one participant commented, "We used to get everything in French and English [Canada's official languages] but now, you know, there's a million other languages out there..." One participant spoke about wanting to have a handout on proper manual dishwashing available in more languages because "...even with the pictures, people still aren't getting it". Some participants also spoke about the difficulty in having food safety resources translated in-house because of the cost of hiring an official translator, and how the cost becomes further prohibitive when resources are needed in more than one language.

#### Meetings with other inspection agencies

Some participants spoke about wanting to meet with inspectors from food inspection agencies at the provincial and federal levels of government, such as OMAFRA and CFIA. These participants spoke about wanting to know about the work and the types of issues with which these agencies were currently involved, because as one participant stated, "Food safety? It's being done by three different agencies: CFIA, OMAFRA, and ourselves...We often don't know what they're doing, and they don't know what we're doing." One participant remembered past inter-agency meetings with CFIA and OMAFRA fondly, and commented on how they led to better relationships between the agencies.

#### Mandatory food safety education for food handlers

Many participants spoke about the benefits of the food handler certification course provided by health units, and felt that the course should be mandatory for all food handlers at food establishments. Some participants discussed how they believed that this implementation would help address many of the food safety issues that they had raised in the focus group discussions because it would provide food handlers with fundamental food safety knowledge. A few participants also discussed how this training is mandatory for employees involved in food preparation in daycares and long-term care facilities, and one participant noted that it may be the reason why they see fewer food handling issues in those facilities.

Some participants also thought that food safety education should be taught in high school as part of the curriculum. For instance, one participant commented, "...make it mandatory that all kids in high school have to take it. It has benefits to your home, everybody prepares food, everybody handles food."

### Workshop topics, format, and length

When asked what topics they would like to see covered in a food safety workshop hosted by the research collaborators, most participants said they would like information about new trends, emerging issues and pathogens, and specialty foods. One participant stated, "Definitely something like current events or current outbreaks. Current things that are going on, like not only...in the province, but maybe beyond that because we import so much stuff from different countries and...some things that they're seeing...may be coming into our province." Some participants also said they wanted to have people from other inspection agencies (i.e., CFIA, OMAFRA) to come and speak about the work that they do.

Many participants said they would like the information to be delivered via expert guest speakers and to be shown examples, when applicable. As an example, one participant expressed wanting an expert on meat products to show them a display of specialty meats so that they can learn what they look like. Many participants also said they would like the workshop to include interactive activities, such as case studies, outbreak scenarios, and table-top exercises, because as one participant said, "involvement from our own experiences is important to incorporate." Participants were unanimous in preferring one full-day workshop session over two half-day sessions.

## Discussion

Valuable information and insights were gathered from the PHIs that participated in our focus groups. The focus groups allowed us to explore, in-depth, the participants' perceptions of the key food safety issues of concern to public health, and their opinions and needs with regards to food safety information resources. We were able to gain this understanding with minimal interviewer influence and were therefore able to gather opinions and insights that were not previously anticipated by the researchers. Thus, while labour-intensive, these focus groups provided us with a detailed level of understanding that could not be gained through quantitative methods alone.

As each focus group consisted of PHIs from a different health unit, it was anticipated that the responses obtained in each of the discussions would vary to some extent. For instance, vermin and pest control was discussed as an important food safety issue in only one of the focus groups, a group in which all of the participants worked in the downtown core of a large, urban city. This city was also the largest city in the Central West region to be represented in the focus groups. The topic of vermin and pest control did not emerge in the other three focus group discussions, which may indicate that it was not one of the foremost food safety issues of concern to these participants. However, it does not preclude that participants in these groups did not regard it as an important issue.

Of the five key food safety issues raised by the focus group participants, three were improper food handling practices: time-temperature abuse, cross-contamination, and inadequate handwashing (including poor personal hygiene). These food handling practices and behaviours are listed among the "Fatal Five" or five major causes of foodborne illness outbreaks in foodservice establishments [12], and were identified by the CDC as being the food handling practices most frequently linked to outbreaks of foodborne illness in the United States between 1993 and 1997 [13]. The implementation of mandatory food safety training for all food handlers and supervisory staff at food service establishments may be fundamental in addressing these issues, and also in reducing the incidence of foodborne illness that may be attributable to these unsafe food handling practices and behaviours.

As set out in the Ontario Public Health Standards 2008 published by the Ministry of Health and Long-Term Care (the provincial agency responsible for food safety), each health unit is required to provide public health programs and services in specified areas, such as food safety, safe water, health hazard prevention and detection, and infectious disease prevention and control. However, the manner in which PHIs are distributed among the different program areas can vary from one health unit to another. That is, PHIs at some health units may specialize in just one program area, while at others they may be involved in a few or all program areas. As a result, a PHI that specializes only in food safety may be more likely to focus on general food handling practices that reduce or eliminate food safety risks, rather than on particular foodborne pathogens. Particular pathogens may be of more concern to PHIs who also work in program areas such as communicable diseases and/or infection control. This may account for the two main categories of responses that were obtained when participants were asked what they perceived to be the key food pathogens of concern (i.e., those who were concerned with particular foodborne pathogens versus those more concerned with safe food handling practices in general). In this study, however, we did not explore which participants specialized in the area of food safety and which did not.

The foodborne pathogens that were listed as key foodborne pathogens of concern (i.e., *Salmonella*, *Campylobacter*, *Escherichia coli*) by some participants are also the top three leading causes of enteric illness reported in Ontario [14]. Furthermore, *Salmonella *and *E. coli *have also been implicated in recent high-profile outbreaks of foodborne illness in the province. In 2005, an outbreak of salmonellosis in Ontario was associated with the consumption of raw and lightly-cooked mung bean sprouts [15] and in the same year, an outbreak of *E. coli *O157:H7 was linked to raw milk sold from unmarked trucks [16].

The intent of this study was not to evaluate the food safety knowledge level of focus groups participants, but to explore the level of confidence they have in their food safety knowledge and to identify any perceived knowledge gaps. Participants across all four focus groups reported confidence with their current knowledge of food safety issues and foodborne pathogens. This confidence may be attributed to the fact that food safety comprises a large component of the work of many PHIs. Additionally, it is a significant part of the curriculum in the post-secondary education required to become a PHI in Canada, and is an area for which many resources are available.

While participants listed a wide variety of online resources for food safety information (e.g., CFIA and CDC websites), these resources were largely not further elaborated on in the discussions. That is, most participants did not comment on what was available at these websites, discuss instances in which they have made use of these websites, nor did they share any opinions they might have about these online resources. This may indicate that while participants are aware that these online resources are available, they may not access them regularly. Further prompting by the moderator may have provided more insight into this matter.

Participants listed a wide range of food safety resources that they would like to have available. A number of themes were shared across focus groups, such as: the need for a central source for food safety information; access to up-to-the-minute food safety information; resources in different languages; and food safety information about specialty foods. Comments made by the participants suggest the need for an online clearinghouse for food safety information. This online clearinghouse would provide PHIs with a central access to food safety information from a variety of sources. Additionally, it could allow PHIs to search one resource for food safety information at their convenience, in a quick and efficient manner, and also allow them to print information and resources as needed. Furthermore, a central access would ensure that the same information and resources are available to all PHIs, and may lead to greater consistency among PHIs and across health units.

Varying interpretations of the requirements of the Food Premise Regulation was cited by participants as a key reason for the lack of consistency occasionally exhibited to food premise operators by PHIs and health units. A potential strategy to help ensure consistency amongst PHIs and across Ontario health units is the development of guidelines to supplement the Food Premise Regulation, designed to provide more explicit guidance to reduce subjectivity of interpretation. Such guidelines could provide a better framework for PHIs and health units to implement the requirements of the Food Premise Regulation in a more cohesive and consistent manner across the province, and ultimately help to minimize some of the confusion experienced by food premise operators.

Specialty foods are becoming increasingly available at food service establishments across Ontario. In 2007, the Food Premises Regulation was amended to allow for additional foods, such as samosas, beef patties, and burritos, to be sold from street food vending carts. As a result of the growing emergence of specialty foods, PHIs are increasingly required to evaluate the safety of foods that may be unfamiliar to them. Participants in the focus groups expressed that a lack of adequate food safety information about specialty foods was a key issue for them and indicated a desire for more information and training in this area. These concerns are similar to those described in an online survey of food safety professionals in the United States, where 67.7% of 334 respondents listed at least one specialty food of concern for which they lacked adequate food safety information [17]. An update to the Food Premise Regulation to contain provisions for specialty foods may also better equip PHIs in ensuring the safety of these foods to the public.

The need for food safety resources in various languages was a common theme that arose in the focus group discussions. This need may be attributed to the fact that nearly 20% of residents in the Central West region reported speaking a mother tongue other than English or French in the 2006 Canadian Census [8]. The development of additional food safety resources in languages other than English may help to alleviate language barrier problems when they exist between PHIs and food handlers or food premise operators, and thus help PHIs to better promote safe food handling practices that reduce the risk of foodborne illness. Given that the three most common mother tongue groups behind English and French are the Chinese languages, Italian, and Spanish [18], the development of food safety resources in these languages may be of highest priority.

### Limitations

The qualitative nature of this study may limit the conclusions that can be drawn from the findings. As the intent of a focus group is to understand, and not infer, we cannot make generalizations based on the results of this study alone as the responses obtained from participants may or may not be representative of the larger population of PHIs. As a result, the authors have conducted a follow-up Internet survey to explore whether the needs and perceptions identified in these focus groups are representative of PHIs across the province of Ontario (unpublished data).

Selection bias may have been introduced to these focus groups because of the volunteer nature of the study. That is, those who volunteered to participate in the focus groups may have given different answers than those who did not volunteer may have given. For example, people with strong opinions could have been more likely to agree to participate than others.

Despite efforts taken on the part of the researchers, it is possible that some participants may not have expressed their own personal opinion as a result of others present at the discussion. That is, some respondents may have adjusted what they said to conform to a popular viewpoint or to a particular group member. Efforts made to minimize such bias included informing participants that there were no "right" or "wrong" answers and that they were encouraged to agree or disagree with any comments. In addition, efforts were made to exclude managers and individuals with supervisory positions from the discussions, to avoid any superior-subordinate relationships that may inhibit discussion. Given the informal and relaxed nature of the focus group discussions, we believe this type of bias was minimal. The extensive amount of information collected, coupled with the repetition of themes, minimize the significance of this limitation.

## Conclusions

This study allowed for an in-depth investigation of participants' perceptions of the key food safety issues of concern to public health, and their opinions and needs with regards to food safety information resources. A key theme that emerged from these focus groups was the need for the development of a centralized, online clearinghouse that would provide PHIs with access to up-to-date and reliable food safety information from a variety of sources. Further research is required to determine whether the opinions and needs identified by the focus groups participants are representative of a larger population of PHIs. The information gathered in this study can provide a basis for the development of resources to meet the specific needs of PHIs involved in protecting and promoting food safety.

## Abbreviations

PHI: Public Health Inspector; CIPHI: Canadian Institute of Public Health Inspectors; OMAFRA: Ontario Ministry of Agriculture, Food and Rural Affairs; CFIA: Canadian Food Inspection Agency; CDC: Centers for Disease Control and Prevention.

## Competing interests

The authors declare that they have no competing interests.

## Authors' contributions

MTP, AQJ, JMS, BJM, and CED contributed to the study design and development of the focus group questioning route. JMS and CED developed the initial research proposal and obtained funding for the work. MTP and AQJ undertook the focus groups and analyzed the data. MTP prepared the first draft of the manuscript. All authors contributed to interpretation of results, have read and approved the final manuscript.

## Pre-publication history

The pre-publication history for this paper can be accessed here:

http://www.biomedcentral.com/1471-2458/10/345/prepub
